# An Essay on the Diagnosis between Erysipelas, Phlegmon and Erythema; with an Appendix Touching the Probable Nature of Puerperal Fever

**Published:** 1819-10-01

**Authors:** 


*An Essay on the Diagnosis between Erysipelas, Phlegmon and Erythema; with an Appendix touching the probable Nature of Puerperal Fever.* By GEORGE HUME WEATHERHEAD, M. D. &c. &c. Octavo, 72 pages, London, 1819.

ERYSIPELAS is one of those *local* demonstrations of a *constitutional* affection which are every day pressing more and more upon our notice, and require to be more deeply studied than they have hitherto been. While we acknowledge the importance of *diagnosis*, we think that Dr. Weatherhead, in confining himself *solely* to this, has greatly abridged the utility of his work, which should have taken in the etiology, and treatment of the disease. How will it be with medical literature, if we must consult one Author on the diagnosis, another on the causes, and a third on the treatment of even a common cutaneous inflammation? In this way we may *levy* taxes upon the professional republic, but they will not be paid; and consequently the general revenue will suffer. On this account we shall attempt to fill up the *lacunæ* which our author has left, and thus endeavour to render this article a useful one to our brethren at large.

The symptomatology of erysipelas will be sufficiently developed when we come to Dr. W's diagnosis of the disease; we shall therefore introduce some observations here on other points of interest.

1. *The Seat of Erysipelas.* Hippocrates speaks in many places of erysipelas of the womb, lungs, throat, &c. But the moderns are pretty well agreed in considering the disease as an inflammation of the cutaneous surface, and especially of the papillary or vasculo-nervous system, spread over the exterior tissue of the cutis vera. Sometimes, however, the inflammation affects the different structures of the skin, and even the cellular membranes that are spread under the surface, and among the muscles. In these latter cases the adjuncts *phlegmonous* or *œdematous* have been coupled with erysipelas. It is to be recollected, however, that this inflammation has an *erratic* tendency, and frequently shifts its seat from the surface of the body to various internal tissues, as the membranes of the brain, the pleura, the peritonæum, &c.

2. *Etiology*. Among the *predisposing* causes of erysipelas we may enumerate what is termed a bilious temperament—plethoric habit; indulgence in fat, oily, and rancid meats; spirituous and vinous liquors; hereditary tendency to the disease; particular kinds of food, as shell-fish, &c. in peculiar constitutions, affording strong evidence of the intimate sympathy existing between the digestive organs and skin.

The *exciting* causes are very numerous, both moral and physical. Violent mental perturbations frequently excite this cutaneous affection. Sudden applications of wet or cold to the body, especially when heated, are among the most common physical causes of erysipelas, together with the suppression of natural or long accustomed evacuations from the system, as the menses, hæmorrhoidal discharge, &c. In short, *idiopathic* erysipelas will be almost always found connected with some internal or constitutional affection.

We now come to Dr. Weatherhead's work, in which the diagnosis is investigated with great labour, and, we should conceive, with more than necessary research. Our author first distinguishes erysipelas from phlegmon. 1. The *tumour* in erysipelas is soft, diffuse, and undefined; in phlegmon, hard, tense, pointed, and circumscribed.—2. In the *latter* the *pain* is lancinating, acute, and throbbing; in erysipelas less violent, somewhat obtuse, and burning. —3. In E. the efflorescence is of a dull red, often inclining to yellow. In P. it is vivid, shining, pellucid.—4. In E. the redness easily vanishes by pressure, and as quickly returns. In P. the redness is more permanent, and less readily disappears under pressure.—5. In P. the thermometrical *heat* exceeds that of E. while the *fever* is less vehement.—6. The inflammation of erysipelas does not fade imperceptibly, as in phlegmon, into the colour of the neighbouring skin, but terminates in an irregular, but well defined margin.—7. Spontaneous vesications are common in E., while their occurrence must be deemed adventitious in P.—8. P. is much benefited by local bleedings. In E.

these are doubtful remedies, our author thinks.—9. P. tends to suppuration, which rarely takes place in simple erysipelas.—10. Erysipelas seems to be a disease, *sui generis,* arising most frequently constitutionally—not convertible into phlegmon.

"I had an infectious erysipelas, that raged for two years in His Majesty's ship, Jalouse, under my care, and in all the cases in which suppuration took place, shortly after the matter was evacuated the pus ceased to be secreted, and the discharge became wholly serous; and as the tumour continued but little diminished after the evacuation of pus, the swelling was evidently owing to that serous effusion attending erysipelas." P. 17.

Into the distinctions between erysipelas and erythema Dr. W. enters still more deeply, and we shall not be able to follow him so minutely as in the above division of the work.

"1. A symptom peculiar to erysipelas, and not to erythema, is the *torpor* and affection of the *brain*, that not uncommonly precedes the efflorescence; thus drowsiness is a symptom of incipient erysipelas, and not of erythema." P. 21.

2. *Delirium* is a frequent attendant upon erysipelas, but not at all, or only casually, on the erythema.—3. Erysipelas creeps, (serpit) erythema not —In the *former*, the efflorescence is fading in one part, while it glows vividly in another —4. There is always danger when erysipelas attacks the head and face, which is not the case in erythema.—5. There is great danger when erysipelas is repulsed from the surface. The symptoms which commonly ensue are, intense fever, prostration of strength, inappetency, dyspnœa, vertigo, sopor, phrenitis, gastritis, gangrene, sphacelus—death. Bonetus relates an instance of repelled erysipelas from the leg, which was followed, in three days, with phrenitis, difficulty of breathing, and, on the eleventh day of the disease, death. Dissection shewed, besides other morbid appearances, the right side of the pleura beset with miliary pustules, and the dura mater spread with similar eruptions. In all these respects erythema bears no similarity.—6. It is rare that the efflorescence precedes the *fever* of erysipelas; although cases may occur wherein the external symptoms are, at first, unaccompanied by fever.

"7. Erysipelas differs from erythema, in being sometimes, nay, when once excited, and under certain circumstances, perhaps in being *always* infectious; that it is sometimes so, is, I think, abundantly established in many instances. I may add, that the impression of its infectious nature is further strengthened in my mind, from having witnessed its influence during a period of two years; and, contrary to what some have asserted, I have seen it infectious under an inflammatory form." P. 27.

8. Erysipelas appears to possess the specific nature of the exanthemata.—9. Erysipelas is always accompanied with more or less *swelling*; whereas in erythema there is no tumefaction.

"Whilst an infectious erysipelas of a phlegmonous type raged in H. M. S. Jalouse, I determined to ascertain if the tumour, even in slight cases, was owing to the effusion of serum into the equal and continuous extension of cellular membrane. I thrust a lancet into the part most tumid and soft, when serum tinged with blood from the puncture flowed from the aperture; by squeezing I could urge the fluid at pleasure, though distant from the orifice, through the puncture, leaving the compressed part wrinkled and flaccid." P. 32.

10. Œdema does not succeed to erythema; nor does erythema pit on pressure.

11. When erysipelas is about to be severe, it is commonly ushered in by, as Cullen expresses it, "Synocha duorum vel trium dierum, plerumque cum somnolentia, sæpe cum delirio," symptoms strongly evincing its origin in constitutional derangement.

In the above we have presented the principal features of distinction which Dr. Weatherhead has drawn up between erysipelas and erythema; the remainder of the volume being taken up with the subject of puerperal fever. We shall therefore, from other sources, and principally from our own experience, add a few observations on the nature and *treatment* of this disease, which we hope will not be considered an unnecessary appendage to Dr. Weatherhead's book.

We have observed, that it is very rare that idiopathic erysipelas makes its appearance on the surface, without some precursory symptoms of constitutional derangement; as wandering pains, lassitude, chills, restlessness, mental anxiety, inappetency, and various other phenomena which usher in the exanthemata, or febrile states of the system in general.

From the second to the fourth day of these premonitory symptoms the local affection appears, with heat, pain, tension, and pricking of some part of the skin. The part then swells a little, and assumes the erysipelatous hue and appearance. The pain of erysipelas is of a peculiar kind, more analogous to that of a burn than any thing else. There is never any *throbbing* of the part.

The constitutional affection, in general, continues, but seldom increases with the eruption; and about the fifth or sixth day a change for the better takes place in both; the cuticle begins to scale, with itching of the part; in short, resolution of the inflammation takes place.

The above is the *regular* march of erysipelas; but the termination is not always so favourable. The disease either assumes a more malignant character in bad habits, or becomes complicated with other affections; as phlegmon, œdema, gangrene, or low typhoid fever, in which latter case it is very dangerous. Its translations to internal organs, especially the envelopes of the brain, are also exceedingly formidable.

M. Renauldin has seen an instance in a female, 50 years of age, where an universal erysipelas covered the whole trunk and extremities, which were intensely red. The patient suffered dreadfully, and could not rest a moment quiet, by night or by day. She felt as if encircled by a devouring fire! Happily this state did not last long; as the disease was soon removed by cooling aperients, and frequently repeated tepid baths.

Relapses of erysipelas are very common; and it often appears *periodically*, vicarious of some suppressed discharge, as of the menses or hæmorrhoids.

*Treatment.* When the disease is mild, there is little to be done, in the way of internal medicines, except gentle aperients, and avoiding cold or wet. But as there is generally some gastric and intestinal or hepatic derangement accompanying the complaint, the exhibition of an *emetic*, at the beginning, is exceedingly useful, though now too much neglected in this country, since *purgation* has so exclusively occupied the minds of the medical public. If the emetic does not also pass briskly downwards, a saline cathartic is next to be administered; and if the pyrexia run high, and the patient is strong, no fear need be entertained of the lancet, especially if the head, or any internal organ, appear to labour in function.

Chance has thrown us much in the way of erysipelas; and we have found that small doses of calomel and antimony, taken every six or eight hours, proved highly advantageous, with or without blood-letting. As for chalky or farinaceous powders, as external applications, they are clumsy contrivances, and never, in our own experience, of any service. Evaporating lotions of the liquor ammon. acet. and mistura camphoræ, have proved most useful in our hands, using them *tepid*, if the inflammation was languid, and disposed to recede; but merely with the chill off, if the patient was strong, and the inflammation in any degree intense. *Cold* saturnine washes, we think, are doubtful—in some cases, dangerous applications. We have seen several translations of the erysipelas from their employment, and numerous instances of this kind are on record.

When erysipelas assumes the *phlegmonous* character, local detractions of blood, especially by leeches, are necessary; and if matter forms, and the cellular membrane between the integuments and muscles becomes affected, as it generally does, free incisions will be found a valuable mean of preventing much mischief in the sequel. We know very well that in private practice this measure is not always, or indeed often permitted, and that it has been condemned, without a trial, by some surgical authorities; but one *positive* evidence is worth a thousand *negative* ones, on practical points of this kind.

In conclusion of this article, we may remark that Dr. Weatherhead's Appendix contains various researches, some facts, and many speculations on the connection, or rather *identity* of puerperal fever and erysipelas. We know that the inflammations of serous and expanded tissues, as of the peritoneum, have naturally many of the erysipelatous characters, as the diffusive march, insidious approach, and dangerous termination; but that they are one and the same disease, remains, we think, to be proved. We do not know that a conviction of their identity would materially bear on our prognosis or treatment of either disease.

We consider Dr. Weatherhead's little work as very creditable to his professional talents and literary character.

---

